# Metabolic improvements of novel microbial fermentation on black tea by *Eurotium cristatum*

**DOI:** 10.3389/fmicb.2023.1287802

**Published:** 2023-12-12

**Authors:** Xiu-ping Wang, Rui-yang Shan, Zhao-long Li, Xiang-rui Kong, Ruo-ting Hou, Hui-ni Wu, Chang-song Chen

**Affiliations:** ^1^Tea Research Institute, Fujian Academy of Agricultural Sciences, Fuzhou, China; ^2^Institute of Animal Husbandry and Veterinary Medicine, Fujian Academy of Agricultural Sciences, Fuzhou, China

**Keywords:** *Eurotium cristatum*, black tea, fermentation, microorganisms, metabolites, interactions

## Abstract

Due to its traditional fermentation, there are obvious limits on the quality improvements in black tea. However, microbial fermentation can provide an abundance of metabolites and improve the flavor of tea. The “golden flower” fungi are widely used in the microbial fermentation of tea and has unique uses in healthcare. To further explore the improvements in black tea quality achieved via microbial fermentation, we used widely targeted metabolomics and metagenomics analyses to investigate the changes in and effects of metabolites and other microorganisms during the interaction between the “golden flower” fungi and black tea. Five key flavor metabolites were detected, the levels of catechin, epigallocatechin gallate, (−)-epicatechin gallate were decreased by different degrees after the inoculation of the “golden flower” fungus, whereas the levels of caffeine and (+)-gallocatechin increased. Botryosphaeriaceae, Botryosphaeriales, Dothideomycetes, Aspergillaceae, Trichocomaceae, and Lecanoromycetes play a positive role in the black tea fermentation process after inoculation with the “golden flower” fungi. D-Ribose can prevent hypoxia-induced apoptosis in cardiac cells, and it shows a strong correlation with Botryosphaeriaceae and Botryosphaeriales. The interaction between microorganisms and metabolites is manifested in tryptophan metabolism, starch and sucrose metabolism, and amino sugar and nucleotide sugar metabolism. In conclusion, the changes in metabolites observed during the fermentation of black tea by “golden flower” fungi are beneficial to human health. This conclusion extends the knowledge of the interaction between the “golden flower” fungi and black tea, and it provides important information for improving the quality of black tea.

## 1 Introduction

Black tea is commonly produced in China, India, Kenya, Sri Lanka, and other countries. It plays a significant role in boosting regional economic growth and battling poverty. Black tea consumption comprises 78% of the world’s tea consumption (Takashi and Isao, 2003; Azapagic et al., 2016; Niranjan et al., 2022). The color and taste of tea mainly depend mainly on non-volatile compounds, such as polyphenols, amino acids, and caffeine, and bioalgae (Zhen, 2002). Catechins accounts for approximately 80 per cent of the total polyphenols; the catechins can be further divided into (+)-gallocatechin, (−)-epigallocatechin, (+)-catechin, (−)-epicatechin, (−)-epigallocatechin-3-gallate, (−)-gallocatechin gallate, (−)-epicatechin-3-gallate, and catechin gallate (Fang et al., 2021). Theanine constitutes around 50% of the total amino acids present. Caffeine is the major purine alkaloid present in tea. These characteristic compounds are responsible for the unique flavors of tea (Zhen, 2002). The fermentation process has a significant impact on the flavor and quality of black tea products. The primary fermentation process of black tea is endogenous enzymatic oxidation; therefore, there is not much capacity for quality improvement, particularly in terms of health benefits and slimming properties (Bancirova, 2010). Microbial fermentation of tea is more important for attaining high-quality flavor and health benefits than endogenous fermentation of black tea. The microbial fermentation process generates large amounts of polyphenol oxidase, cellulase, and pectinase, which considerably enhance the health benefits, esthetic appeal, and weight reduction of tea (Tang et al., 2022; Zhang et al., 2022). The expansion of the black tea processing chain using microbial flora is, therefore, essential to enhancing the quality of black tea.

The production of Fu-brick tea and other dark teas relies heavily on the “golden flower” fungi. The “golden flower” fungi were identified as *Eurotium cristatum* (Du et al., 2019), they can metabolize and transform phenolic compounds such as catechins in tea. Being among the primary components of tea, these metabolites have no effects on normal cells while having antimutagenic, anticancer, and tumor cell-inhibitory effects (Oguni et al., 1988; Mukhtar et al., 1992; Katiyar et al., 1993; Lea et al., 1993; Du et al., 2019). Therefore, these metabolites are beneficial to the health of the human body. According to some studies, adding the “golden flower” fungi to finished black tea after fermentation can raise theophylline levels and enhance the black tea’s quality (Yu et al., 2015). This study confirms the potential of the “golden flower” fungi to improve the growth and processing of black tea products. Unfortunately, the precise molecular mechanism underlying the interaction between the “golden flower” fungi and black tea has not been investigated. To enhance the flavor and health benefits of black tea and to encourage the growth of the black tea industry, it is important to investigate the changes in the microbial population occurring during the interaction between the “golden flower” fungi and black tea.

Various symbiotic bacteria, including *Bacillus* bacteria, must work together with the “golden flower” fungi for the fermentation of tea (Xiang et al., 2022). Determining the symbiotic functional microorganisms involved in fermentation by the “golden flower” fungi is, therefore, essential for improving this process and ensuring a consistent output of metabolites. The community composition and organization of the aforementioned symbiotic bacteria are no longer a mystery because of the widespread use of macrogenetic sequencing techniques (Huang et al., 2019). The composition and function of the secondary metabolites are also frequently investigated using high-throughput next-generation sequencing technologies (Huang et al., 2019; Fan et al., 2021). With the help of these two techniques, we can clarify the corresponding relationship between functional microorganisms and functional metabolites in the interaction between the “golden flower” fungi and black tea. The joint analysis of the metagenome and the metabolome can establish the logical relationships among microbes, metabolites, and phenotypes. The research into how various microorganisms affect the secondary metabolites of tea has been severely constrained by the absence of such studies, which hinders the promotion and advancement of the fermentation process using the “golden flower” fungi.

In order to investigate the changes, effects, and interactions of microorganisms and metabolites during black tea fermentation after the addition of the “golden flower” fungi, this study selected finished black tea, inoculated it with the fungus, and used widely targeted metabolomics and metagenomic analysis techniques. By utilizing the “golden flower” fungi, this study enhances the quality, flavor, and health benefits of black tea. It also contributes favorably to the advancement of deep processing technology for black tea and the expansion of tea goods.

## 2 Materials and methods

### 2.1 Sample collection

The fresh leaves processed into black tea used in this research were from “Fuxuan,” a new variety of high-quality tea tree bred by the Fujian Provincial Academy of Agricultural Sciences. The “golden flower” fungi were isolated and obtained from Anhua dark tea. The pre-*E. cristatum* samples refer to untreated black tea, while the post-*E. cristatum* samples are black tea samples that were irradiated with two rounds of food-grade cobalt-60 radiation and then inoculated with the “golden flower” fungi at a concentration of 10^8–9^ pfu/mL. The samples were cultured at a temperature of 26°C and a relative humidity of 50% for 5 days, followed by low-temperature drying. Both groups of samples underwent comprehensive targeted metabolomic profiling and metagenomic sequencing.

### 2.2 Total antioxidant capacity assay

Take 20 mg of freeze-dried samples, add 100 microliters of frozen PBS solution, thoroughly crush the pulp, release antioxidant, centrifuge approximately 12,000× *g* for 5 min at 4°C, and take up the supernatant. The antioxidant capacity was measured by using the Total Antioxidant Capacity Assay Kit with the FRAP method (Beyotime Biotechnology, S0116).

### 2.3 Widely targeted metabolomics analysis

The freeze-dried samples were crushed with a mixer mill for 30 s at 60 Hz. After precisely weighing 10 mg aliquots of individual samples, 500 μL of extract solution (methanol/water = 3:1, precooled at −40°C, containing internal standard) was added. After 30 s of vortex mixing, the samples were centrifuged at 12,000 rpm (RCF = 13,800× *g*, *R* = 8.6 cm) for 15 min at 4°C. After the supernatant was carefully filtered through a 0.22 μm microporous membrane, the samples were analyzed by Allwegene Company (Beijing). The mobile phase A was 0.1% formic acid in water, and the mobile phase B was acetonitrile. The column temperature was set to 40°C. The autosampler temperature was set to 4°C, and the injection volume was 2 μL. A Sciex QTrap 6,500+ (Sciex Technologies) was applied for assay development. Typical ion source parameters were as follows: ion spray voltage, +5,500/−4,500 V, curtain gas: 35 psi, temperature: 400°C, ion source gas 1, 60 psi; ion source gas 2, 60 psi; DP, ±100 V.

### 2.4 Metabolome data processing and analysis

SCIEX Analyst Workstation Software (Version 1.6.3) was employed for MRM data acquisition and processing. MS raw data (.wiff) files were converted to the .txt format using msConvert. The R program and database were applied for peak detection and annotation (Smith et al., 2006; Kuhl et al., 2012; Zhang et al., 2015). In the ion chromatogram, the target compounds showed symmetrical chromatographic peaks, and the chromatographic separation of the target compounds was well achieved. After data processing, orthogonal partial least squares-discriminant analysis (OPLS-DA) and principal component analysis were performed on metabolite data before and after inoculation, and the results were visualized by scatter plots (Mi et al., 2019; Yang et al., 2020). Differentially accumulated metabolites (DAMs) between the pre-*E. cristatum* and post-*E. cristatum* groups were determined according to *t-*test value of *p* <0.05 and VIP >1. Lastly, the KEGG pathway database was used to enrich the metabolic pathways of DAMs, and R was used to visualize the results.

### 2.5 DNA extraction and metagenomics analysis

The total DNA of the tea samples was extracted using the CTAB/SDS method. The purity and quality of the genomic DNA were checked on 1% agarose gels, and the DNA concentration was precisely quantified using a Qubit fluorometer. DNA sequencing libraries were deep-sequenced on the Illumina Novaseq PE150 platform at Allwegene Company (Beijing). The quality of the raw data was assessed using fastp (Chen et al., 2018), and then these high-quality reads were assembled to contigs using MEGAHIT (Li et al., 2015)(parameters: kmer_min = 47, kmer_max = 97, step = 10).^1^ Open reading frames (ORFs) in contigs were identified using Prodigal (Hyatt et al., 2010).^2^ A nonredundant gene catalog was constructed using CD-HIT (Fu et al., 2012).^3^

### 2.6 Metagenomics data processing and analysis

The taxonomic annotation was conducted using Diamond^4^ (Buchfink et al., 2015) against the Kyoto Encyclopedia of Genes and Genomes database^5^ with an e-value cutoff of 1 × 10^−5^. The annotation of antibiotic resistance genes was conducted using Diamond^6^ (Buchfink et al., 2015) against the CARD database^7^ with an e-value cutoff of 1 × 10^−5^. To generate bar plots illustrating the composition of dominant species at various taxonomic levels in each sample, the R language was utilized. LEfSe analysis was conducted to identify biomarkers across groups. KEGG metabolic pathways were analyzed to detect the distinctions and changes in the functional genes of metabolic pathways and in the protein functions of bacterial communities among different groups. Combining the abundance information for unigenes, the relative abundance of antibiotic resistance genes (ARGs) was calculated for significant differential analysis. Canonical correlation and correlation analyses were used to analyze the correlation between signature microorganism communities and these detected metabolites.

## 3 Results

### 3.1 Total antioxidant capacity assay

We tested the antioxidant properties of the black tea before and after inoculation with the “golden flower” fungi in order to evaluate the tea’s chemical characteristics (Table 1). Antioxidant capacity increased from 0.46 to 0.59 μmol/mL after inoculation.

**Table 1:** The total antioxidant capacity in post-*E. cristatum* and pre-*E. cristatum* samples.

	Pre-*E.cristatum*	Post-*E.cristatum*	Log_2_ fold change	*p*-value
	(μmol/mL)	(μmol/mL)	(Pre-*E.cristatum* vs. Post-*E.cristatum*)	
Total antioxidant capacity	0.46 ± 0.005	0.59 ± 0.004	−0.35	0.00001***

### 3.2 Quantitative analysis of flavor-related metabolites

In order to assess the flavor changes in black tea after inoculation with the “golden flower” fungi, quantitative analysis was performed on 10 key flavor metabolites: caffeine, theanine, (−)-epicatechin, (−)-epicatechin gallate, epigallocatechin gallate, catechin, epigallocatechina, gallocatechina, gallocatechine gallate, and catechine gallate. Among these metabolites, five were detected in black tea before and after inoculation (Table 2). Specifically, the content of catechin, epigallocatechin gallate, (−)-epicatechin gallate were decreased by different degrees after the inoculation of the “golden flower” fungus. In contrast, the content of (+)-gallocatechin and caffeine increased after inoculation.

**Table 2:** The differences in the contents of 10 flavor-related metabolites in post-*E. cristatum* and pre-*E. cristatum* samples.

Name	Formula	Pre-*E.cristatum*	Post-*E.cristatum*	Log_2_ Fold Change	*p*-Value
				(Pre-*E.cristatum* vs. Post-*E.cristatum*)	
Epigallocatechin gallate	C_22_H_18_O_11_	4.677 ± 0.70	2.200 ± 0.21	1.09	0.004**
(−)-Epicatechin gallate	C_22_H_18_O_10_	15.510 ± 0.62	8.890 ± 0.64	0.80	0.0002***
Catechin	C_15_H_14_O_6_	0.222 ± 0.05	0.132 ± 0.02	0.75	0.048*
(+)-Gallocatechin	C_15_H_14_O_7_	0.005 ± 0.00	0.183 ± 0.16	−5.20	0.117
Caffeine	C_8_H_10_N_4_O_2_	416.258 ± 11.76	542.496 ± 22.90	−0.38	0.001***
Theanine	C_7_H_14_N_2_O_3_	/	/		
(−)-Epicatechin	C_15_H_14_O_6_	/	/		
Epigallocatechin	C_15_H_14_O_7_	/	/		
Gallocatechin gallate	C_22_H_18_O_11_	/	/		
Catechin gallate	C_22_H_18_O_10_	/	/		

### 3.3 Metabolic characteristics of tea samples

**Figure 1:**
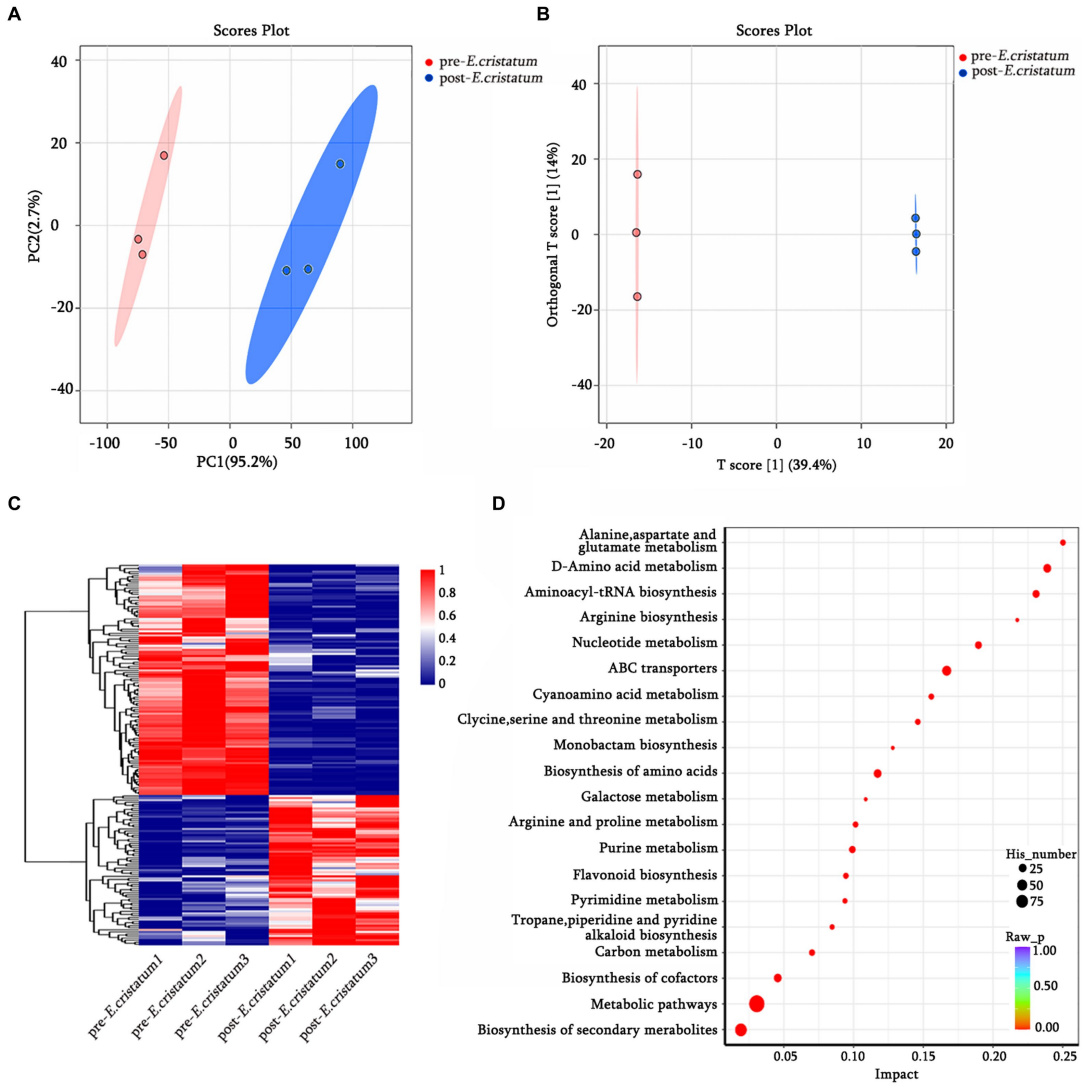
The qualitative and functional analyses of metabolome data for pre-*E. cristatum* and post-*E. cristatum* samples. **(A)** The scatter plot of PCA scores of pre-*E. cristatum* and post-*E. cristatum* samples. **(B)** The OPLS-DA model of pre-*E. cristatum* and post-*E. cristatum* samples. **(C)** The DAM clustering heat map comparing samples. **(D)** The top 20 KEGG pathways identified in the enrichment analysis of DAMs.

The data we obtained by conducting comprehensive targeted metabolomic analysis on black tea samples before and after inoculation with the “golden flower” fungi were further subjected to a series of multivariate pattern recognition analyses. Principal component analysis (PCA) revealed the first component of the two groups of tea leaves (Figure 1A) with an *R^2^X* value of 95.2%, indicating good discrimination between the two groups. Orthogonal partial least squares discriminant analysis (OPLS-DA) showed significant differences between the two types of tea (Figure 1B), with scores from both groups falling within the 95% confidence interval. Through KEGG annotation, a total of 156 metabolites were identified (Figure 1C). Within the KEGG annotation, the 156 differentially regulated metabolites were mapped to 80 pathways (Figure 1D), with the majority enriched in metabolic pathways, the biosynthesis of secondary metabolites, ABC transporters, D-amino-acid metabolism, and other material metabolic processes.

### 3.4 Composition of bacterial and fungal communities in pre-*E. cristatum* and post-*E. cristatum* samples

**Figure 2:**
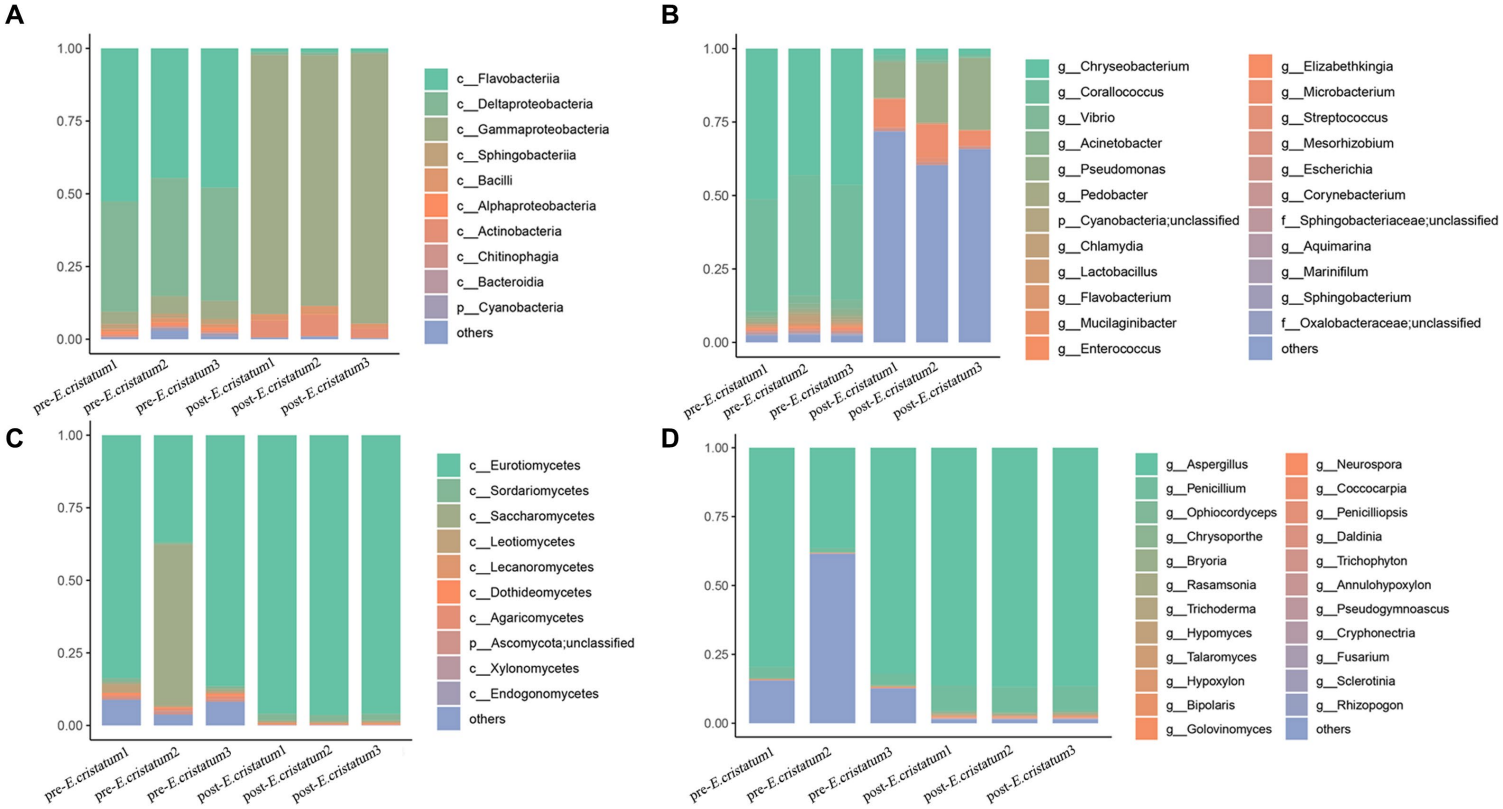
The diverse species composition of pre-*E. cristatum* and post-*E. cristatum* samples. **(A)** The species composition at the bacterial class level. **(B)** The species composition at the fungal class level. **(C)** The species composition at the bacterial genus level. **(D)** The species composition at the fungus genus level.

After obtaining the data, we annotated the species at different classification levels in the black tea samples and analyzed the changes in the species composition before and after inoculation with the “golden flower” fungi. The annotation of the bacterial species showed that, at the class level (Figure 2A), the proportions of Flavobacteriia and Deltaproteobacteria decreased significantly from 47 and 38 to 1% after inoculation with the “golden flower” fungi. The proportion of Gammaproteobacteria, in contrast, increased dramatically from 5 to 89%, making them the dominant bacteria. At the genus level (Figure 2C), *Chryseobacterium* and *Corallococcus* showed significant decreases after inoculation with the “golden flower” fungi, decreasing from 46 and 40% to 2 and 13%, respectively. The proportion of *Pseudomonas*, however, increased after inoculation with the “golden flower” fungi. The annotation of the fungal species showed that, at the class level (Figure 2B), Eurotiomycetes strains were present at the highest proportions both before and after inoculation with the “golden flower” fungi, with the post-inoculation proportion increasing to 96%. At the genus level (Figure 2D), *Aspergillus* strains were present at the highest proportions both before and after inoculation, with the post-inoculation proportion increasing to 86%.

### 3.5 Screening analysis of signature microorganisms and differential gene analysis

**Figure 3:**
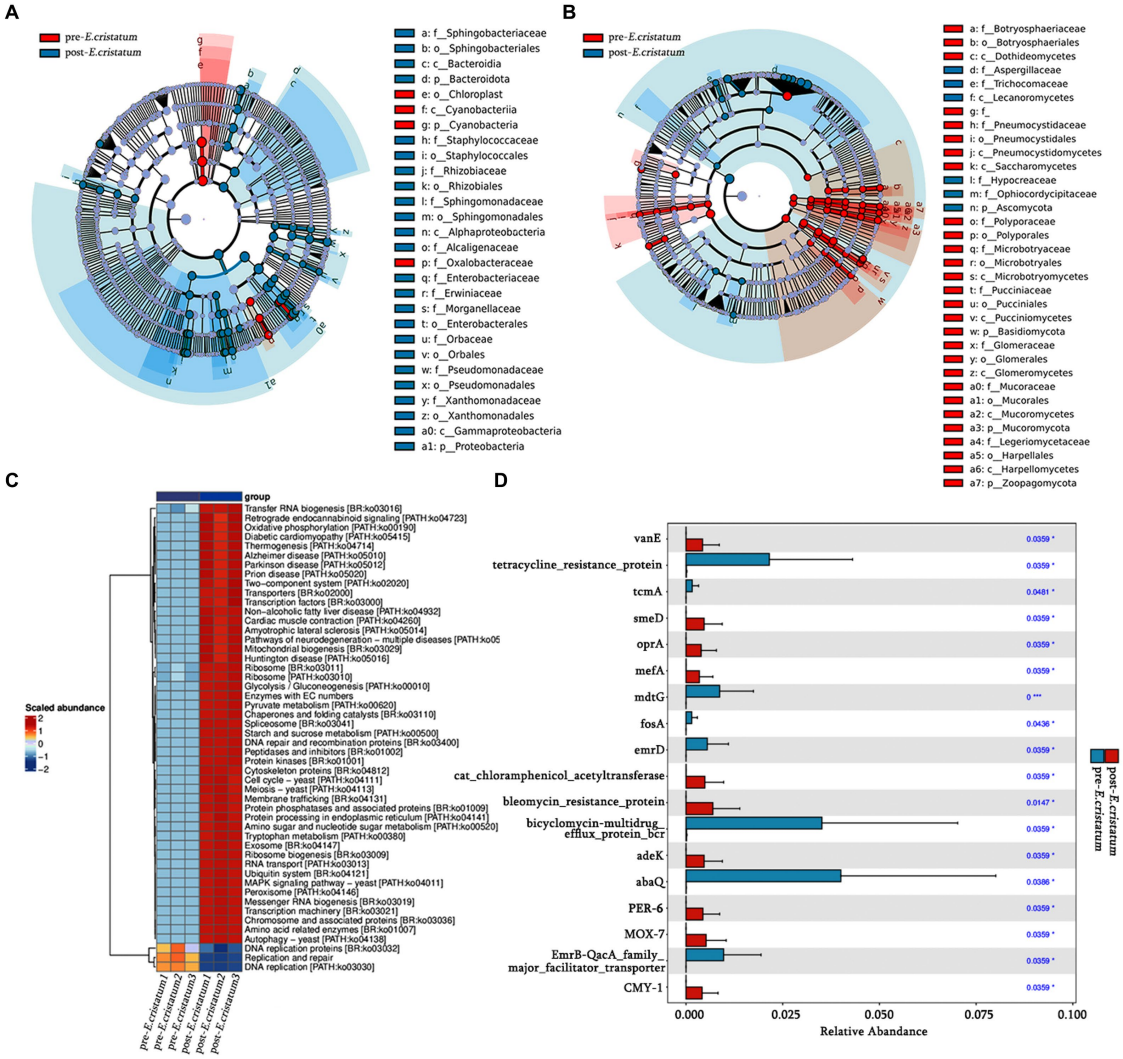
The signature microorganisms and bacterial pathway annotations. **(A)** Characteristic bacterial LEfSe analysis. **(B)** Characteristic fungal LEfSe analysis. **(C)** KEGG pathways enriched in bacteria. **(D)** Histogram of significantly different resistance genes.

LefSe analysis was performed to identify signature bacteria and fungi and further analyze the microbial community. Sphingobacteriaceae, Sphingobacteriales, and Bacteroidia were identified as characteristic bacterial communities after inoculation (Figure 3A), whereas Aspergillaceae, Trichocomaceae, and Lecanoromycetes were the characteristic fungal communities after inoculation (Figure 3B). Chloroplast, Cyanobacteriia, and Cyanobacteria were the characteristic bacterial communities before inoculation (Figure 3A), whereas Botryosphaeriaceae, Botryosphaeriales, and Dothideomycetes were the characteristic fungal communities before inoculation (Figure 3B). After inoculation with the “golden flower” fungi, the specific fermentation environment of black tea became unfavorable for the survival of the pre-inoculation signature microorganisms, leading to their disappearance.

Based on KEGG annotation, the differences in the functional genes of the microbial community in black tea samples before and after inoculation with the “golden flower” fungi are demonstrated (Figure 3C). After inoculation with the “golden flower” fungi, the abundance of many KEGG pathways increased, including tryptophan metabolism, starch and sucrose metabolism, and amino sugar and nucleotide sugar metabolism. Analysis of the significantly differentially expressed antibiotic resistance genes (Figure 3D) showed that most of the resistance genes, such as *abaQ* and bicyclomycin-multidrug efflux protein_bcr, were significantly increased in black tea after inoculation with the “golden flower” fungi.

### 3.6 Comprehensive analysis of microorganisms and metabolites

**Figure 4:**
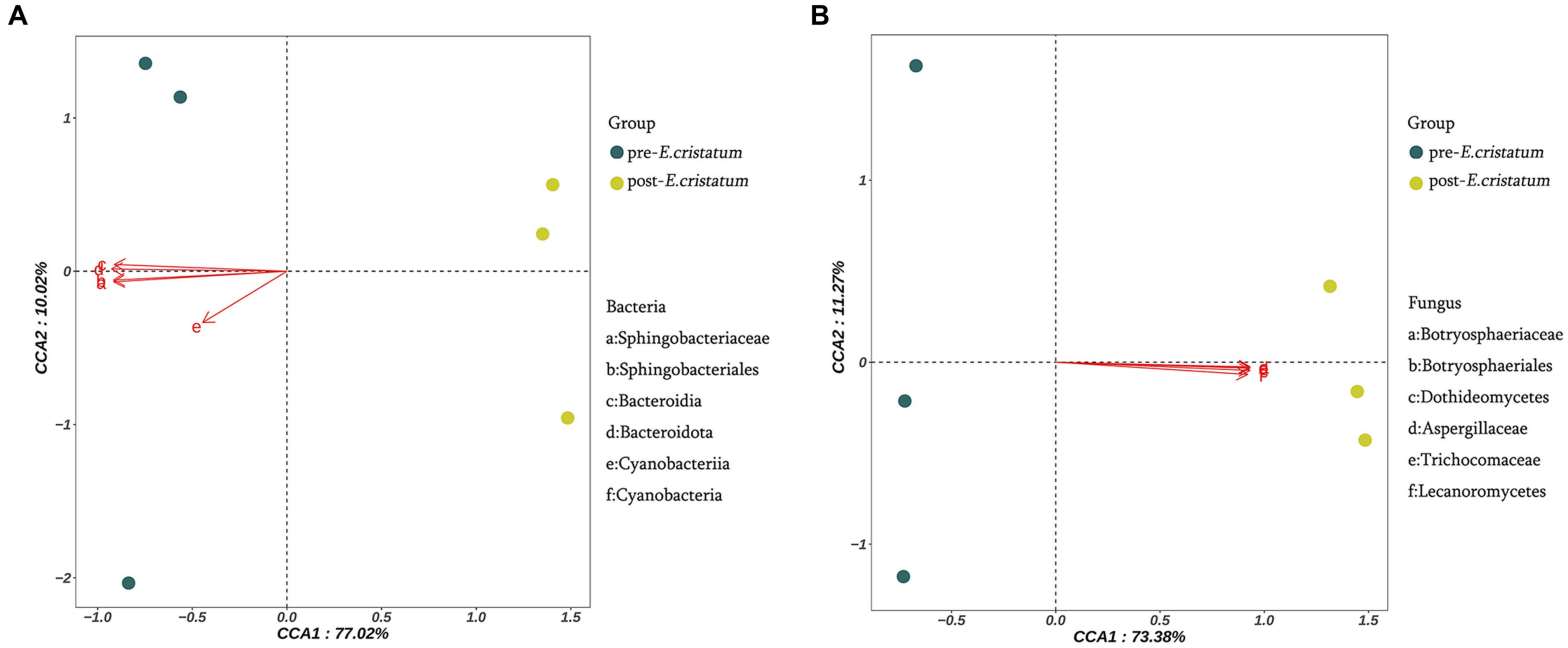
Canonical correlation analysis of microorganisms and metabolites. **(A)** Canonical correlation analysis of signature bacterial communities and metabolites. **(B)** Canonical correlation analysis of signature fungal communities and metabolites.

The canonical correlation analysis of the effects of signature microorganisms was performed on the distribution of metabolites before and after the inoculation with the “golden flower” fungi (Figure 4). Sphingobacteriaceae, Sphingobacteriales, Bacteroidia, Bacteroidota, Cyanobacteriia, and Cyanobacteria had a negative impact on the fermentation of black tea after inoculation with the “golden flower” fungi, with Bacteroidota playing the most significant role (Figure 4A). Additionally, Botryosphaeriaceae, Botryosphaeriales, Dothideomycetes, Aspergillaceae, Trichocomaceae, and Lecanoromycetes had a positive impact on the fermentation of black tea after inoculation (Figure 4B). The changes in five flavor metabolites may be associated with these microbial communities, with fungi such as Botryosphaeriaceae, Botryosphaeriales, Dothideomycetes, Aspergillaceae, Trichocomaceae, and Lecanoromycetes assisting in fermentation by the “golden flower” fungi. Correlation analysis (Supplementary Table S1) revealed that d-ribose was the metabolite most strongly correlated with Botryosphaeriaceae and Botryosphaeriales; 2-picolinic acid was the metabolite most strongly correlated with Dothideomycetes; l-arginine was the metabolite most strongly correlated with Aspergillaceae and Trichocomaceae; D-sorbitol was the metabolite most strongly correlated with Lecanoromycetes.

**Figure 5:**
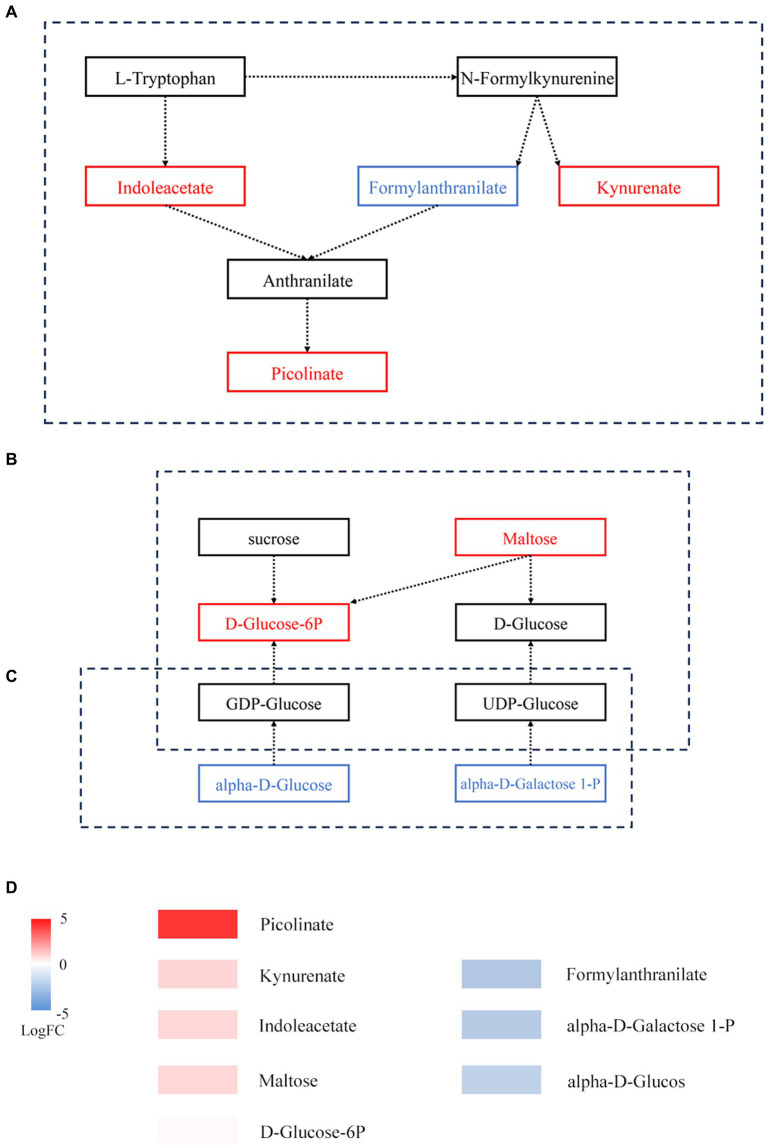
Metabolic pathways of DAMs before and after fermentation. **(A)** Tryptophan metabolism. **(B)** Starch and sucrose metabolism. **(C)** Amino sugar and nucleotide sugar metabolism. The box represents metabolites; red represents upregulated metabolites, while blue represents downregulated metabolites. **(D)** Expression of DAMs involved in panels **(A–C)**.

On the basis of KEGG annotation (Figure 5), the abundance of tryptophan metabolism, starch and sucrose metabolism, and amino sugar and nucleotide sugar metabolism pathways increased. Metabolite enrichment analysis of these pathways revealed 9 DAMs. In tryptophan metabolism, 3 DAMs, namely, indoleacetate, kynurenate, and picolinate, were upregulated, while 1 DAM, formylanthranilate, was downregulated. In starch and sucrose metabolism, 2 DAMs, namely, maltose and D-glucose-6P, were upregulated. In amino sugar and nucleotide sugar metabolism, 3 DAMs, namely, alpha-D-glucose, galactose-1-P, and D-arabinose, were downregulated.

## 4 Conclusion

As distinctive microorganisms, fungi such as those in the Botryosphaeriaceae, Botryosphaeriales, Dothideomycetes, Aspergillaceae, Trichocomaceae, and Lecanoromycetes families may be used for the fermentation of black tea. D-Ribose, 2-picolinic acid, L-arginine, and D-sorbitol are metabolites that have strong relationships with particular bacteria. The “golden flower” fungi have long been regarded as one of the most significant microbial constituents in tea, but the beneficial symbiotic bacteria and metabolites that are connected to them also invite additional research to uncover the causal linkages among the various biota engaged in tea fermentation. Enhancing black tea quality and streamlining the production process can be accomplished by determining the microbial succession and interaction of metabolites that affect the biochemical composition of black tea.

## 5 Discussion

Fermented microorganisms and metabolites give tea many beneficial qualities (Zhen, 2002; Tang et al., 2022), but few studies have explored the mechanisms of the effect of “golden flower” tea on black tea. In this study, widely targeted metabolomics revealed significant changes in the metabolites; five of these are key flavor metabolites closely linked to the taste and effect of tea. Furthermore, metagenomic analysis techniques revealed fluctuations in microbial clusters; 6 types of fungi are characteristic microorganisms because of their great changes.

The functions of tea-related compounds are diverse, and most research has focused on the functional contributions of metabolites (Someya et al., 2002; Wu et al., 2023). Quantitative analysis of 7 flavor metabolites has been explored in other research (Wu et al., 2023), and proved that these flavor metabolites have an important role to play in the taste and health effects of Fu-brick tea. Compared with this study, there have been changes in the levels of caffeine and gallocatechin after black tea’s inoculation with “golden flower” fungi (Table 1). Gallocatechin has antioxidant functions and protective effects against diseases such as cancer and heart disease (Someya et al., 2002). Caffeine relaxes smooth muscle of the bronchi, making it of value for the treatment of asthma and the bronchospasm of chronic bronchitis (Lin et al., 2022). In addition, the various metabolites were predominantly enriched in metabolic pathways, secondary metabolite production, ABC transporters, D-amino acid metabolism, and other material metabolic activities after the inoculation with the “golden flower” fungi. Theanine, caffeine, and catechins, which are the secondary metabolites found in tea, are significant contributors to the distinctive flavors of tea (Fang et al., 2021). A potential target for regulating immunity and infection is amino acid metabolism (Tomé, 2021).

Our observations were largely supported by earlier research linking the “golden flower” fungi to the biochemical makeup of tea. For example, few studies on Fu-brick tea have explored the alteration of microbial communities in the fermentation process (Xiang et al., 2022). In the current study, after the inoculation of black tea with “golden flower” fungi, the trend of changes in the contents of Eurotiomycetes and Dothideomycetes is consistent with the trend of change after the fermentation of Fu-brick tea (Xiang et al., 2022), Eurotiomycetes gradually becomes the dominant class, while the content of Dothideomycetes significantly decreases. The microbial composition of black tea changes after inoculation. For example, there is a decrease in Flavobacteriia and Deltaproteobacteria, and an increase in *Penicillium*, Lecanoromycetes, and Gammaproteobacteries. Some *Penicillium* spp. can produce l-asparaginase, and this enzyme is being employed for acute lymphoblastic leukemia and lymphosarcoma therapies (De Carvalho et al., 2021). Lecanoromycetes can be consumed as a herbal supplement, as well as being a component of tea and foods (Xu et al., 2018; Song et al., 2022). Gammaproteobacteria, one of the important microorganisms in the fermentation process, is a signature microorganism in Fu-brick tea. These changes play a key role in the health-related qualities of black tea. After inoculation with “golden flower” fungi, the specific fermentation environment of black tea is not conducive to the survival of microorganisms such as Flavobacteriia and Deltaproteobacteria and causes their reduction or disappearance.

There is a strong link between certain metabolites, such D-ribose, and specific bacteria. In hypoxic cardiac cells, D-ribose has been demonstrated to inhibit apoptosis (Caretti et al., 2013). L-arginine administration over an extended period was shown to ameliorate cardiovascular disease symptoms (Boger, 2014). Within a specific range, D-sorbitol can improve the body’s absorption of vitamin B (Herbert et al., 1959). Tryptophan metabolism, starch and sucrose metabolism, and amino sugar and nucleotide sugar metabolism all depend on the interplay of microbes and metabolic byproducts. The entire immunological homeostasis depends heavily on tryptophan metabolism (Le Floc’h et al., 2011). The products of amino sugar and nucleotide sugar metabolism can enter starch and sucrose metabolism (Figure 5C). In starch and sucrose metabolism, D-glucose-6P is produced from starch under the catalysis of beta-fructofuranosidase, and the significant increase in D-glucose-6P and maltose may be caused by the accelerated rate at which starch is degraded. A variety of fungi play a role in promoting fermentation by the “golden flower” fungi in black tea (Figure 4B). These compounds may serve as a source of energy during fermentation by the “golden flower” fungi. Additionally, we also analyzed the expression of antibiotic resistance genes; *abaQ*, sodium chloride, certain antibiotics, and multidrug resistance genes can yield antibacterial and antiviral effects (Clark et al., 1998; Pérez-Varela et al., 2018; Kato et al., 2019; Baron et al., 2020).

Artificial inoculation with “golden flower” fungi is a new approach to improving tea products. Studies have shown that adding “golden flower” fungi to finished black tea can increase the level of theophylline (Yu et al., 2015). However, these studies mainly focused mainly on the taste and sensory qualities of tea. In this study, we focused not only on metabolic changes, but also on the appearance of the fungus and bacteria, thus providing a comprehensive understanding of the molecular mechanisms in the fermentation process. Furthermore, our research explored the common roles of metabolites and microorganisms. It is critical to find any microbes and metabolites that could support the growth of the “golden flower” fungi and develop black tea. However, the further effects of dominant strains and distinctive metabolites on black tea are still unclear. Future research on these prevalent strains and metabolites may help expand the black tea industry.

## Data availability statement

The original contributions presented in the study are included in the article/Supplementary material, further inquiries can be directed to the corresponding author.

## Author contributions

X-pW: Data curation, Writing – original draft. R-yS: Methodology, Resources, Writing – original draft. Z-lL: Methodology, Writing – original draft. X-rK: Data curation, Software, Writing – original draft. R-tH: Writing – original draft. H-nW: Writing – original draft. C-sC: Writing – review & editing.

## Footnotes

^1^https://github.com/voutcn/megahit, version 1.1.2

^2^ https://github.com/hyattpd/prodigal/wiki

^3^http://www.bioinformatics.org/cd-hit/, version 4.6.1

^4^http://www.diamondsearch.org/index.php, version 0.8.35

^5^http://www.genome.jp/keeg/, version 94.2

^6^http://www.diamondsearch.org/index.php, version 0.8.35

^7^https://card.mcmaster.ca/home, version 3.0.9
